# Low preoperative albumin-globulin score predicts favorable survival in esophageal squamous cell carcinoma

**DOI:** 10.18632/oncotarget.8868

**Published:** 2016-04-20

**Authors:** Fei Zhang, Peng Sun, Zhi-qiang Wang, De-shen Wang, Yun Wang, Dong-sheng Zhang, Feng-hua Wang, Jian-hua Fu, Rui-hua Xu, Yu-hong Li

**Affiliations:** ^1^ Collaborative Innovation Center for Cancer Medicine, Guangzhou, Guangdong, 510060, People's Republic of China; ^2^ State Key Laboratory of Oncology in South China, Guangzhou, Guangdong, 510060, People's Republic of China; ^3^ Department of Medical Oncology, Sun Yat-sen University Cancer Center, Guangzhou, Guangdong, 510060, People's Republic of China; ^4^ Guangdong Esophageal Cancer Institute, Guangzhou, Guangdong, 510060, People's Republic of China; ^5^ Department of Thoracic Surgery, Sun Yat-sen University Cancer Center, Guangzhou, Guangdong, 510060, People's Republic of China

**Keywords:** esophageal squamous cell carcinoma, albumin-globulin score, albumin/globulin ratio, survival

## Abstract

This study retrospectively investigated the prognostic significance of the preoperative albumin-globulin score (AGS) and albumin/globulin ratio (AGR) in esophageal squamous cell carcinoma (ESCC). A cohort of 458 newly diagnosed ESCC patients who underwent radical esophagectomy in Sun Yat-sen University Cancer Center (Guangzhou, China) between January 2006 and December 2010 were selected into this study. The optimal cut-off value was identified to be 45.6 g/L, 26.9 g/L and 1.30 for albumin (ALB), globulin (GLB) and AGR in terms of survival, respectively. Patients with low ALB levels (< 45.6 g/L) and high GLB levels (≥ 26.9 g/L) were assigned an AGS of 2, those with only one of the two abnormalities were assigned an AGS of 1, and those with neither of the two abnormalities were assigned an AGS of 0. Univariate survival analysis showed that low AGS (0) was significantly associated with favorable disease free survival (DFS) [hazard ratio (HR), 0.635; 95% confidence interval (CI), 0.441–0.914; *P* = 0.015] and overall survival (OS) (HR, 0.578; 95% CI, 0.387–0.862; *P* = 0.007), and it remained an independent predictor for OS (HR, 0.630; 95% CI, 0.418–0.952; *P* = 0.028), but not for DFS (HR, 0.697; 95% CI, 0.479–1.061; *P* = 0.060) in multivariate models. High AGR (≥ 1.30) was also correlated with favorable DFS (HR, 0.626; 95% CI, 0.430–0.910; *P* = 0.014) and OS (HR, 0.622; 95% CI, 0.422–0.916; *P* = 0.016) in univariate analysis, but it failed to be an independent prognostic indicator for DFS (HR, 0.730; 95% CI, 0.494–1.078; *P* = 0.114) or OS (HR, 0.759; 95% CI, 0.507–1.137; *P* = 0.181) by multivariate analysis. Low preoperative AGS could serve as a valuable and convenient biochemical marker to predict favorable long-term survival in ESCC patients.

## INTRODUCTION

Esophageal cancer is the eighth most common cancer and fourth cause of cancer-related death worldwide, with more than 480,000 new cases and 400,000 deaths each year, half of which occur in China [1–2]. The main pathological subtypes include esophageal squamous cell carcinoma (ESCC) and esophageal adenocarcinoma (EAC). EAC remains the major subtype in some Western countries, whereas ESCC is the predominant histological type in certain regions of Asian countries [3–4]. Although great progress has been made in the treatment of ESCC in the past decades, the prognosis still remains poor. Thus, identification of promising prognostic factors contributing to the risk classification and clinical management of such patients could improve their long-term survival.

The American Joint Committee on Cancer (AJCC), the Union for International Cancer Control (UICC) tumor-node- metastasis (TNM) staging system, and the histopathological parameters are the most important prognostic indicators [5–7]. Additionally, some inflammation-based prognostic indicators, such as the modified Glasgow Prognostic Score (mGPS) and the neutrophil-lymphocyte ratio (NLR) have also emerged as potential prognostic factors in ESCC [8–10]. Moreover, some researches have revealed that systemic inflammation response and the proinflammatory cytokines in the tumor microenvironment could contribute to tumor progression, distant metastasis, suppression of adaptive immunity, and less response to chemotherapy, thus leading to poor survival [11–14].

Albumin (ALB) and globulin (GLB), the two major components of serum proteins, have been confirmed to be involved in the systemic inflammatory process. Hypoalbuminemia in cancer patients is not only an indicator of poor nutritional status but also relates to chronic inflammation [15], and low ALB levels could be the result of cytokine-induced immune suppression [16]. Moreover, increased levels of GLB could serve as markers of chronic inflammation response and reflect a cumulative exposure of various proinflammatory cytokines [12]. Previous studies have demonstrated that hypoalbuminemia was associated with impaired survival in ESCC patients [17–18]. However, no studies have been performed to explore the cumulative effect of both ALB and GLB on ESCC patients. Therefore, the purpose of this present study was to access the effect of the preoperative albumin-globulin score (AGS) and albumin/globulin ratio (AGR) on long-term survival among ESCC patients.

## RESULTS

### Patients’ baseline characteristics

There were 345 men (75.3%) and 113 women (24.7%) with a median age of 59.0 years (ranged, 20.0– 88.0 years). Of these, 40 (8.7%) were stage I, 219 (47.9%) were stage II and 199 (43.4%) were stage III. Forty (8.7%) patients were with tumors located at upper esophagus, while there were 285 (62.2%) and 133 (29.1%) patients with tumors located at middle and lower esophagus respectively. And the numbers of patients with poorly/not differentiated, moderately differentiated and well differentiated tumors were 117 (25.6%), 237 (51.7%) and 104 (22.7%) respectively. The clinicopathologic characteristics of the included 458 patients were demonstrated in Table 1.

**Table 1:** Baseline characteristics of 458 patients with ESCC

Variables	No. of patients	Percentage (%)
Age (years)		
> 60	194	42.4
≤ 60	264	57.6
Gender		
Male	345	75.3
Female	113	24.7
Tumor lacation		
Lower	133	29.1
Middle	285	62.2
Upper	40	8.7
Tumor length (cm)		
< 5	247	53.9
≥ 5	211	46.1
Differentiation		
Well	104	22.7
Moderate	237	51.7
Poor/Undifferentiated	117	25.6
T stage		
T1	42	9.2
T2	74	16.2
T3	307	67.0
T4	35	7.6
N stage		
N0	242	52.8
N1	119	26.0
N2	76	16.6
N3	21	4.6
TNM stage		
I	40	8.7
II	219	47.9
III	199	43.4
Alcohol		
No	301	65.7
Yes	157	34.3
Smoking		
No	169	36.9
Yes	289	63.1
mGPS		
0	369	80.6
1	81	17.7
2	8	1.7
WBC count (k/cm^3^)^#^	7.4 ± 2.4	
NEC count (k/cm^3^)^#^	4.5 ± 2.0	
LYM count (k/cm^3^)^#^	2.1 ± 0.7	
NLR^#^	4.14 ± 1.73	

ESCC, esophageal squamous cell carcinoma; TNM, tumor-node-metastasis; mGPS, modified Glasgow Prognostic Score; WBC, white blood cell; NEC, neutrophil cell; LYM, lymphocyte; NLR, neutrophil-lymphocyte ratio, ^#^, presented as mean ± standard deviation.

### Correlation between AGS, AGR, and clinicopathological parameters

The optimal cut-off value was identified to be 45.6 g/L, 26.9 g/L and 1.30 for ALB, GLB and AGR in terms of survival, respectively. The results demonstrated that low AGS (0) was correlated with less advanced age, tumor length, tumor-node-metastasis (TNM) stage, depth of invasion, less alcohol consumption and high neutrophil-lymphocyte high NLR. No significant correlation was identified between AGS and gender, tumor location or other parameters (Table 2). Whereas high AGR (≥ 1.30) was more frequently seen in male gender, patients with less advanced tumor length, less peripheral leukocyte and neutrophil count. There was no significant difference in the distribution of age, tumor location, TNM stage or other variables between AGR groups.

**Table 2:** Correlation between AGS, AGR, and clinicopathologic parameters in 458 ESCC patients

Variables	No. of patients		*P* value	No. of patients		*P* value
	AGR			AGS		
	High (≥ 1.30)	Low (< 1.30)		High (1–2)	Low (0)	
Age (years)			0.299			0.001^*^
> 60	173	21		176	18	
≤ 60	244	20		209	55	
Gender			0.041^*^			0.103
Male	320	25		284	61	
Female	97	16		101	12	
Tumor lacation			0.176			0.276
Lower	125	8		109	24	
Middle	254	31		239	46	
Upper	38	2		37	3	
Tumor length (cm)			0.002^*^			< 0.001^*^
< 5	235	12		193	54	
≥ 5	182	29		192	19	
Differentiation			0.967			0.387
Well	95	9		87	17	
Moderate	215	22		204	33	
Poor/Undifferentiated	107	10		94	23	
T stage			0.237			0.003^*^
T1	40	2		29	13	
T2	69	5		61	13	
T3	279	28		260	47	
T4	29	6		35	0	
N stage			0.253			0.811
N0	223	19		200	42	
N1	106	13		103	16	
N2	71	5		64	12	
N3	17	4		18	3	
TNM stage			0.464			0.01^*^
I	38	2		27	13	
II	201	18		186	33	
III	178	21		172	27	
Alcohol			0.378			0.044^*^
No	271	30		261	40	
Yes	146	11		124	33	
Smoking			0.068			0.519
No	148	21		145	24	
Yes	269	20		240	49	
WBC count (k/cm^3^)^#^	7.3 ± 2.2	9.0 ± 3.3	0.001^*^	7.5 ± 2.4	7.1 ± 1.9	0.548
NEC count (k/cm^3^)^#^	4.4 ± 1.9	5.7 ± 3.0	0.002^*^	4.6 ± 2.1	4.2 ± 1.5	0.184
LYM count (k/cm^3^)^#^	2.1 ± 0.6	2.3 ± 1.0	0.191	2.1 ± 0.7	2.2 ± 0.6	0.124
NLR^#^	4.12 ± 1.68	4.31 ± 2.18	0.797	4.07 ± 1.71	4.51 ± 1.80	0.023^*^

AGS, albumin-globulin score; AGR, albumin/globulin ratio; #, presented as mean ± standard deviation.

* *P* < 0.05.

### Value of AGS and AGR in predicting the long-term survival in ESCC

The median follow-up time was 46.8 months (ranged, 1.0–106.0 months). Two hundred and forty-five patients died from ESCC before the end of the follow-up period. Median DFS and OS for the whole population were 35.9 months (95% CI, 25.3–46.4 months) and 57.0 months (95% CI, 39.7–74.4 months), respectively.

**Figure 1:**
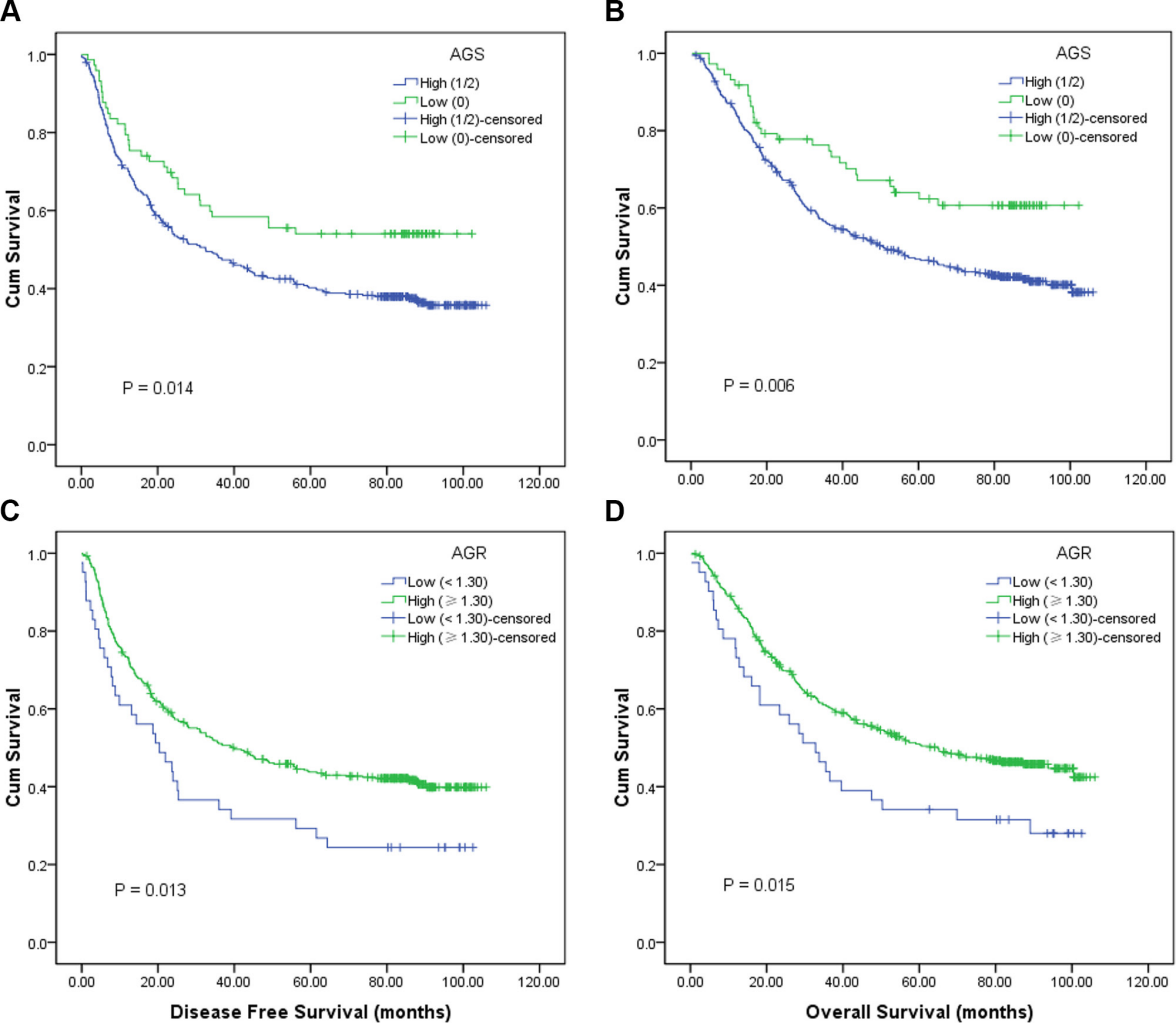
Kaplan-Meier survival curves of 458 esophageal squamous cell carcinoma patients (**A**) disease-free survival (DFS) (*P* = 0.014); (**B**) overall survival (OS) (*P* = 0.006) stratified by their preoperative albumin-globulin score (AGS); C, DFS (*P* = 0.013); D, OS (*P* = 0.015) stratified by their preoperative albumin/globulin ratio (AGR). (with log-rank test).

Cox univariate models showed that low AGS was significantly associated with favorable DFS (HR, 0.635; 95% CI, 0.441–0.914; *P* = 0.015; Figure 1A) and OS (HR, 0.578; 95% CI, 0.387–0.862; *P* = 0.007; Figure 1B). Tumor length (< 5/≥ 5 cm), T stage (T1–2/T3–4), lymph node status (Negative/Positive), TNM stage (I–II/III), smoking history (Yes/No) and alcohol consumption (Yes/ No) were other significant prognostic variables identified by univariate analysis. On multivariate analysis, low AGS remained to be an independent predictor for OS (HR, 0.630; 95% CI, 0.418–0.952; *P* = 0.028), but not for DFS (HR, 0.697; 95% CI, 0.479–1.061; *P* = 0.060) (Tables 3 and 4).

**Table 3:** Univariate and multivariate analysis of DFS in 458 ESCC patients

Variables	Univariate			Multivariate		
	HR	95% CI	*P* value	HR	95% CI	*P* value
Age (years)						
> 60	1		0.830			NI
≤ 60	0.974	0.766–1.238				
Gender						
Male	1		0.044^*^	1		0.850
Female	0.743	0.557–0.992		0.96	0.629–1.464	
Tumor location						
Lower	1		0.878			NI
Middle	1.122	0.712–1.766				
Upper	1.037	0.469–2.291				
Tumor length (cm)						
< 5	1		0.004^*^	1		0.344
≥ 5	1.419	1.118–1.800		1.128	0.879–1.450	
Differentiation						
Moderate/Well	1		0.171			NI
Poor/Undifferentiated	1.211	0.920–1.594				
T stage						
T1–T2	1		< 0.001^*^			NI
T3–T4	1.587	1.414–1.781				
Lymph node status						
Negative	1		< 0.001^*^			NI
Positive	1.748	1.543–1.981				
TNM stage						
I–II	1		< 0.001^*^	1		< 0.001^*^
III	2.333	1.833–2.969		2.121	1.650–2.727	
Alcohol						
No	1		0.013^*^	1		0.242
Yes	1.364	1.067–1.744		1.185	0.892–1.574	
Smoking						
No	1		0.030^*^	1		0.586
Yes	1.325	1.028–1.707		1.112	0.759–1.631	
WBC count (k/cm^3^)						
< 7.1	1		0.058			NI
≥ 7.1	1.26	0.992–1.600				
NEC count (k/cm^3^)						
< 4.1	1		0.064			NI
≥ 4.1	1.253	0.987–1.591				
LYM count (k/cm^3^)						
< 2.0	1		0.991			NI
≥ 2.0	1.001	0.789–1.270				
NLR						
< 3.8	1		0.041^*^	1		0.301
≥ 3.8	0.78	0.615–0.990		0.879	0.689–1.122	
ALB (g/dL)						
≥ 45.6	1		< 0.001^*^			NI
< 45.6	1.671	1.259–2.220				
GLB (g/dL)						
< 26.9	1		0.715			NI
≥ 26.9	1.046	0.821–1.334				
AGR						
< 1.30	1		0.014^*^	1		0.114
≥ 1.30	0.626	0.430–0.910		0.730	0.494–1.078	
AGS						
1–2	1		0.015^*^	1		0.060
0	0.635	0.441–0.914		0.697	0.479–1.061	
mGPS						
1–2	1		0.027^*^	1		0.918
0	0.729	0.550–0.964		0.984	0.726–1.335	

DFS, disease-free survival; HR, hazard ratio; CI, confidence interval; ALB, albumin; GLB, globulin; NI, not included.

* *P* < 0.05.

**Table 4:** Univariate and multivariate analysis of OS in 458 ESCC patients

Variables	Univariate			Multivariate		
	HR	95% CI	*P* value	HR	95% CI	*P* value
Age (years)						
> 60	1		0.445			NI
≤ 60	1.103	0.858–1.419				
Gender						
Male	1		0.066			NI
Female	0.753	0.557–1.019				
Tumor location						
Lower	1		0.878			NI
Middle	1.122	0.712–1.766				
Upper	1.037	0.469–2.291		
Tumor length (cm)						
< 5	1		0.015*	1		0.824
≥ 5	1.364	1.062–1.753		1.030	0.793–1.338	
Differentiation						
Moderate/Well	1		0.179			NI
Poor/Undifferentiated	1.219	0.913–1.626				
T stage						
T1-T2	1		< 0.001^*^			NI
T3-T4	1.822	1.492–2.225				
Lymph node status						
Negative	1		< 0.001^*^			NI
Positive	1.739	1.526-1.980				
TNM stage						
I–II	1		< 0.001^*^	1		< 0.001^*^
III	2.448	1.897–3.159		2.211	1.696–2.881	
Alcohol						
No	1		0.003^*^	1		0.048^*^
Yes	1.474	1.139–1.907		1.356	1.003–1.832	
Smoking						
No	1		0.043^*^	1		0.689
Yes	1.318	1.009–1.722		1.066	0.778–1.461	
WBC count (k/cm^3^)						
< 7.1	1		0.161			NI
≥ 7.1	1.197	0.931–1.539				
NEC count (k/cm^3^)						
< 4.1	1		0.216			NI
≥ 4.1	1.172	0.912–1.506				
LYM count (k/cm^3^)						
< 2.0	1		0.645			NI
≥ 2.0	0.943	0.734–1.211				
NLR						
< 3.8	1		0.008^*^	1		0.090
≥ 3.8	0.711	0.553–0.914		0.801	0.619–1.036	
ALB (g/dL)						
≥ 45.6	1		< 0.001^*^			NI
< 45.6	1.824	1.342–2.478				
GLB (g/dL)						
< 26.9	1		0.608			NI
≥ 26.9	0.935	0.722–1.210				
AGR						
< 1.30	1		0.016^*^	1		0.181
≥ 1.30	0.622	0.422-0.916		0.759	0.507–1.137	
AGS						
1–2	1		0.007^*^	1		0.028^*^
0	0.578	0.387–0.862		0.630	0.418–0.952	
mGPS						
1–2	1		0.004^*^	1		0.323
0	0.655	0.490–0.875		0.854	0.642–1.168	

OS, overall survival.

* *P* < 0.05.

Additionally, high AGR was also correlated with favorable DFS (HR, 0.626; 95% CI, 0.430– 0.910; *P* = 0.014; Figure 1C) and OS (HR, 0.622; 95% CI, 0.422– 0.916; *P* = 0.016; Figure 1D) in univariate analysis, but it was not an independent prognostic indicator for DFS (HR, 0.730; 95% CI, 0.494–1.078; *P* = 0.114) or OS (HR, 0.759; 95% CI, 0.507–1.137; *P* = 0.181) in multivariate analysis (Tables 3 and 4).

### Exploratory analysis

We further performed an exploratory analysis to evaluate the synergistic prognostic value of TNM stage and AGS for ESCC patients. Patients were classified into four groups based on the combination of TNM stage and AGS. Group A corresponded to those stage I/II patients with an AGS of 0; B corresponded to stage I/ II patients with an AGS of 1/2; C corresponded to stage III patients with an AGS of 0 and D corresponded to stage III patients with an AGS of 1/2. Kaplan-Meier survival analysis revealed that the estimated median OS for them were not reached, 100.4 months, 41.0 months and 27.2 months, respectively (*P* = 0.019).

## DISCUSSION

It has been increasingly recognized that systemic inflammation is associated with poor prognosis in patients with cancer [11–14]. Recent studies have demonstrated that an increased systemic inflammatory response before surgery is an independent prognostic factor of survival following resection of esophageal cancer [7, 19–20]. And the mGPS, NLR, as well as other inflammation-based prognostic scores have been established to predict the survival of ESCC [8–10]. As two major markers of systemic chronic inflammation, the combination of ALB and GLB have showed potential predictive effect on survival among several malignancies [21–23]. Unfortunately, we failed to identify the preoperative AGR as an independent prognostic indicator for ESCC patients in the present study. Interestingly, we found great differences in the distribution of patients’ age, tumor length, T stage, TNM stage and alcohol consumption between AGS groups. Besides, low AGS was significantly correlated with increased NLR. Furthermore, multivariate COX regression model revealed that AGS was a significant independent predictor for the long-term survival in ESCC patients. To the best of our knowledge, the present study was the first one demonstrating the prognostic value of preoperative AGS in ESCC.

The results demonstrated that high AGS was significantly associated with more aggressive behavior. However, no correlation was found between AGS and lymph node metastasis, which was similar to a recent study. Arigami et al. suggested that mGPS was not correlated with lymph node metastasis (*P* = 0.185) in ESCC patients who underwent esophagectomy with lymphadenectomy [24]. And the underlying potential mechanism merited further experimental investigation.

GPS and mGPS are two most famous prognostic markers in patients with various malignancies including ESCC [7–8, 25–26]. Previous studies have suggested that mGPS could serve as novel predictors of chemoradiotherapy responsiveness and independent prognostic indicators for long-term survival in patients with inoperable ESCC [25–26]. However, mGPS failed to be a significant prognostic factor in the present study. Neither did Arigami et al. showed mGPS as an independent prognostic indicator in operable ESCC [24]. In addition, Wei et al. indicated that as the majority of the ESCC patients were classified in the group of a score of 0, mGPS just could not distinguish the survival differences of most of the patients, thus, the combination of mGPS and other prognostic indexes would better distinguish the survival differences [10]. Moreover, treatment approaches and tumor biological behavior could also strongly influence the prognostic significance of mGPS in ESCC patients.

We compared the prognostic impact of AGS, TNM stage and alcohol consumption in the present study. The log-rank test indicated significant survival differences among ESCC patients stratified by AGS, as well as TNM stage and alcohol consumption. Furthermore, the results demonstrated that the HR for AGS was higher than that for alcohol drinking (1.731 vs. 1.356), but lower than that for TNM stage (1.731 vs. 2.211). Therefore, the AGS was found to be superior to alcohol consumption, but inferior to TNM stage for predicting OS in patients with ESCC. In addition, the exploratory analysis indicated that the combination of TNM stage and AGS had more accuracy in predicting the long-term survival in ESCC.

The main limitations of this study were the retrospective single-center design, the limited study cohort and lack of measurement of other specific inflammatory markers such as cytokine levels. Despite these limitations, our study demonstrated that low preoperative AGS could serve as a valuable and convenient biochemical marker to predictfavorable long-term survival of ESCC patients. And together with other significant prognostic factors, it could also assist clinicians with better individualization of their therapeutic approach based on the risk stratification. However, further larger prospective studies are needed to validate this finding and to investigate other prognostic indicators in ESCC patients.

## MATERIALS AND METHODS

### Patients

We selected a consecutive cohort of 560 ESCC patients who underwent radical esophagectomy at the Department of Thoracic Surgery, Sun Yat-sen University Cancer Center between January 2006 and December 2010. Exclusion criteria were as follows: patients who received preoperative chemotherapy and/or radiotherapy, patients who had concurrent liver disease and those who received immunosuppressive therapy (e.g. recent steroid exposure). Additionally, to eliminate the influences of non-cancer diseases on prognosis, patients with chronic inflammatory diseases including autoimmune disorder and infection were also excluded. Therefore, a group of 458 cases matched the inclusion and exclusion criteria and were selected in our finial analysis.

### Treatment and follow up

The surgical approaches included both left and right transthoracic esophagectomy. Only tumors originated from the lower and middle sections of the thoracic esophagus underwent a left transthoracic esophagectomy with a longitudinal resection margin more than 5 cm from the tumor. The tumor-bearing esophagus was resected en bloc together with the adjacent tissues. At least a two-field lymph node dissection, including standard, extended, or total dissection of the thoracic and abdominal lymph nodes, was performed in all patients. Adjuvant treatment options were determined based on tumor stage, doctor's selection and patient's desire. Chemotherapy was typically utilized with a two-drug regimen of platinum-based drugs for 4–6 cycles. Radiotherapy was delivered to the anastomosis, supraclavicular, and mediastinal lymphatics, with a total dosage of 46–64 Gy.

All patients were followed-up postoperatively every 3 months for the first 2 years, every 6 months in the third year and yearly thereafter. Physical examination, upper gastrointestinal endoscopy, ultrasonography, tumor marker and computed tomography were regularly evaluated during the follow-up period.

### Clinical and laboratory parameters

Patients’ baseline characteristics including demographic parameters, tumor histology and stage, and laboratory variables were retrospectively reviewed and collected from the electronic medical records. The AJCC/UICC TNM staging system (the 7th edition) was applied to classify the tumor stage. The tumor length was defined as the long diameter measured with the general post-operative pathological specimens. The tumor locations were classified into upper esophagus, middle esophagus and lower esophagus. And the degree of differentiation was categorized into poorly/not differentiated, moderately differentiated and well differentiated.

All blood samples were collected from the forearm vein between 7:00 to 9:00 a.m. within three days prior to surgery, and were immediately sent for analysis. The serum levels of ALB, GLB and other variables were tested by an automatic biochemical analyzer (Hitachi 7600, Japan). Patients with low ALB levels (< 45.6 g/L) and high GLB levels (≥ 26.9 g/L) were assigned an AGS of 2, those with only one of the two abnormalities were assigned an AGS of 1, and those with neither of the two abnormalities were assigned an AGS of 0. As for mGPS, patients with C-reactive protein (CRP) < 10 mg/L were allocated a score of 0, those with both CRP > 10 mg/L and ALB > 35 g/L were allocated a score of 1, while patients with both CRP > 10 mg/L and ALB < 35 g/L were allocated a score of 2.

### Ethics statement

All included patients were asked to provide written informed consent for their information to be recorded and used in our cancer registry. The study was approved by the independent ethics committees at Sun Yat-sen University Cancer Center and was performed in accordance with the ethical standards of the World Medical Association Declaration of Helsinki.

### Statistical analysis

Chi-square or Mann-Whitney *U* test was utilized to examine the differences of baseline and clinicopathologic characteristics between groups. DFS was calculated from the date of diagnosis to local recurrence/distant metastasis or to the last date of follow-up, OS was the time interval from the date of diagnosis to death from ESCC or to the last date of follow-up. Survival curves were estimated using the Kaplan-Meier method, and differences were compared with the log-rank test. Cox proportional hazards models were used for univariate and multivariate analysis to determine hazard ratios (HRs) for variables respecting to DFS and OS. HRs with 95% confidence intervals (CIs) and two-sided *P* value were reported. The optimal cutoff value for certain variables was determined with the method established by Jan Budczieset al. at http://molpath.charite.de/cutoff/ [27]. All statistical analyses were performed with SPSS 17.0 (SPSS Inc., Chicago, IL, USA). And a two-sided *P* value of less than 0.05 was considered to be statistically significant.
